# Geo-visual integration of health outcomes and risk factors using excess risk and conditioned choropleth maps: a case study of malaria incidence and sociodemographic determinants in Ghana

**DOI:** 10.1186/s12889-019-6816-z

**Published:** 2019-05-06

**Authors:** Sylvester Dodzi Nyadanu, Gavin Pereira, Derek Ngbandor Nawumbeni, Timothy Adampah

**Affiliations:** 1 ECHO Research Group International, P. O. Box Fl 424, Aflao, Ghana; 2 School of Public Health, Curtin University, Perth, Australia; 3 Honorary Research Associate, Telethon Kids Institute, Perth, Australia

**Keywords:** Sociodemographic determinants, Excess risk maps, Conditioned choropleth maps

## Abstract

**Background:**

Recently, exploratory spatial data analysis is for problem solving, hypothesis generation and knowledge construction. Unless geographically weighted regression, sophisticated spatial regression models best control spatial heterogeneity in outcomes and the associated risk factors but cannot visually display and identify areas of the significant associations. The under-utilised excess risk maps (ERMs) and conditioned choropleth maps (CCMs) are useful to address this issue and simplify epidemiological information to public health stakeholders without much statistical backgrounds. Using malaria and sociodemographic determinants in Ghana as case study, this paper applied ERM and CCM techniques for identification of areas at elevated risk of disease-risk factor co-location.

**Method:**

We computed and smoothed mean district-specific malaria incidences for the period 2010 to 2014 as a function of sociodemographic determinants. The spatial distribution of malaria was investigated through global and local spatial autocorrelations, and the association with sociodemographic risk factors evaluated with bivariate correlations. ERMs and CCMs were produced for the statistically significant risk factors.

**Results:**

The incidence of malaria increased over time with cluster locations detected, predominantly at the northern parts but later few spread to the middle parts of the country. Our results suggested that with respect to sociodemographic determinants, district variations in malaria rates might be explained by inequalities in seven sociodemographics, including an unexpected significant negative association with non-religious affiliation. The sociodemographics had positive spatial autocorrelations, exhibited statistically significant interactions and the strongest was observed in urbanisation-basic education correlation (*p*< 0.01, *r* = +0.969). The ERMs and CCMs specifically identified locations with lower or higher than expected rates with respect to particular risk factor(s) where improving risk factor(s) such as employment-to-population ratio in rural areas, basic education could have cascade effects to reduce the expected malaria incidence in endemic areas.

**Conclusion:**

Ghana remains malaria hyperendemic region with district-level spatial heterogeneity. Significant association between malaria and sociodemographics was detected and the ERMs and CCMs geo-visually pinpointed locations of these significant associations. To complement sophisticated spatial regression models, the easily interpretable ERMs and CCMs could be used to specify where disease-risk factor associations are significant, simplifying complex spatial epidemiological information for efficient public health administration.

## Background

Spatial statistical methods offer means to exploit space-time information to detect and quantify patterns in public health data and to investigate the degree of association between putative risk factors and diseases that vary geographically [1]. Various elementary exploratory data analysis methods and geo-visualisation techniques are commonly used in health research to detect patterns, isolate outliers, and identify clusters. These methods produce maps that are used in health research to display geographical patterns in the distribution of health outcomes but do not link the spatial patterns directly with associated risk factors [2, 3]. Geographically Weighted Regression (GWR) is commonly used to examine the spatial pattern in regression coefficients and residuals but the conventional spatial regressions such as spatial lag and spatial error models best establish significant multivariate spatial associations between health outcome and the associated risk factors but cannot visually display where these associations are significant. The constructions of maps that control for risk factors has not yet been well adopted [2], yet have great potential to simultaneously identify locations of elevated risk and disease outcome that exceed expectation [3]. In addition to the GWR, two underutilized methods that yield easily interpretable results and can fully integrate spatially varying health outcomes and covariates are excess risk maps (ERMs) and conditioned choropleth maps (CCMs). The excess risk map detects the concentration of the occurrences of the incidence of the disease and risk factors at a particular place as compared to the overall average incidence [4]. Conditioned choropleth maps (CCMs) control for suspected risk factors by partitioning the study cohort into homogeneous groups based on two risk factors [2]. The CCMs produce multiple micromaps arranged in a two-way panel that allow location-specific comparisons of the association between two covariates and the disease outcome [2, 4–6]. Despite their usefulness in public health administration for solving problems, hypothesis generation, and knowledge construction, excess risk and conditioned choropleth maps [2] have been under-utilised in spatial epidemiology. Unlike sophisticated regression models, ERM and CCM techniques provide means to visually display geographic patterns in the disease–risk factor interactions, simplifying complex spatial statistics into easily interpretable format for health policy makers and public health practitioners without much expertise in spatial epidemiology. The aim of this study was to demonstrate the value of excess risk maps (ERMs) and conditioned choropleth maps (CCMs) in the identification of areas at elevated risk, using malaria and sociodemographics as a case study.

Malaria remains the leading cause of morbidity and mortality in sub-saharan Africa [7, 8]. The distribution of malaria within a geographical area can vary greatly between districts, villages and households [9]. Malaria is hyper-endemic and perennial in all parts of Ghana, accounting for 44% of outpatient attendance, 13% of all hospital deaths and 22% of mortality among children less than five years [10]. The major factors influencing the geographic distribution of malaria include climatic, environmental, land-use, land-cover, physical and socio-economic factors [11], which modify behaviour of the malaria vector [12]. Sociodemographic factors associated with malaria transmission and epidemics include household construction, house type, household overcrowding, personal protection measures against mosquito bites, ethnic groups, education, lower income or unemployment, family living standards, knowledge and awareness about malaria [9, 13, 14]. Sociodemographic characteristics can also influence the effectiveness, understanding, acceptance and the usage level of intervention programmes and disease progression. It has also been shown that culturally-varying perceptions and knowledge about malaria varies among communities which can be integrated into traditional health education messages to enhance effectiveness of public health efforts to control malaria in Ghana [15]. In this study, we used ERMs and CCMs to visualise the influence of sociodemographic risk factors on the geographical distribution of malaria at local level of public health administration in Ghana. Despite the significant burden of malaria in Ghana this has not yet been undertaken previously using routine clinically diagnosed and nationwide coverage malaria data and sociodemographic determinants and therefore has great potential to inform malaria management.

## Methods

### Study population and setting

**Fig. 1:**
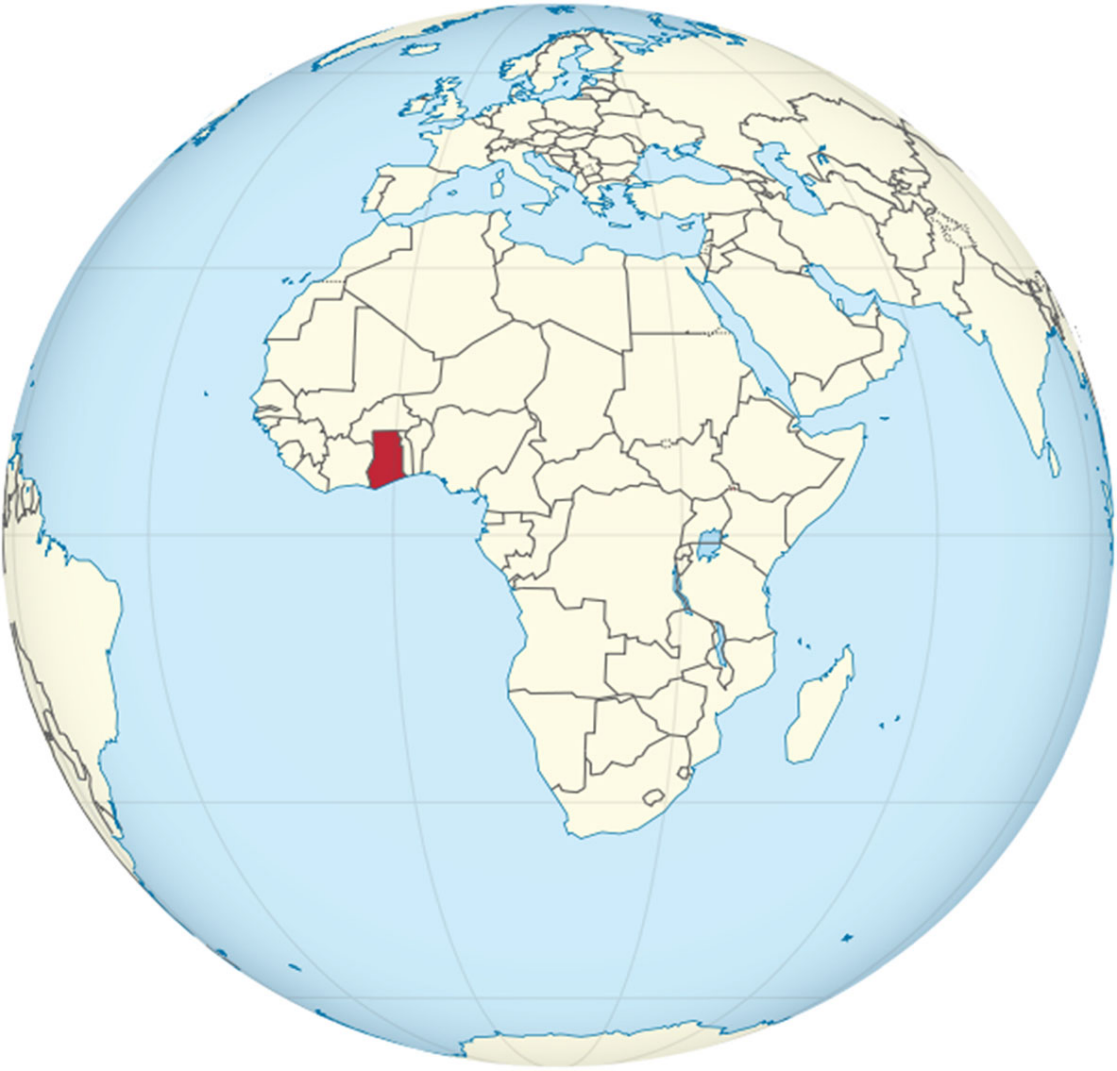
The globe showing Ghana (red) within Africa centered (Retrieved from https://commons.wikimedia.org/wiki/File:Ghana_on_the_globe_(Africa_centered).svg

Ghana, located in the Sub-Saharan African (Figure 1), has a population of more than 27 million people and a population density of 113persons/km^2^. Approximately 51% of the populations are residing in urban centres according to the 2010 census [16]. Ghana has ten administrative regions, subdivided into 170 districts on which 2010 census was conducted, and has approximately 75 ethnic groups with different socio-cultural practices [16, 17]. This study was conducted on the 170 districts using malaria incidence for the 2010 – 2014 period.

### Data sources and variables

Clinically diagnosed malaria cases for outpatient visits at all health facilities in Ghana during the study period 2010–2014, were obtained from the Centre for Health Information and Management (CHIM) within the Ghana Health Service (GHS). Routinely, clinical diagnoses of malaria are based on parasitological microscopy and/or rapid diagnostic test (undertaken at public and private hospitals, clinics and health centres) but mostly by rapid diagnostic test at Community Health Planning Services programme (CHPS) zones in accordance with World Health Organization criteria. The CHPS programme improves coverage of malaria ascertainment for underserved communities and villages in rural areas by using trained community health nurses to render basic clinical and public health services, including diagnosis and treatment of malaria. The entire population of Ghana is at risk of malaria since malaria is endemic in all parts of the country with seasonal variations. Shapefiles for the 170 local health administrative districts were obtained from the Survey and Mapping Divisions, Accra and the Geomatic department of KNUST. Data were available nationally at the district level. Sociodemographic characteristics were obtained from the 2010 Population and Housing Census (PHC) which had complete population coverage on the 170 districts, providing information relating to the various aspects of the populations and households. The district-level proportions (expressed as percentage of the total) of the socio-demographic factors used in the study were described concisely as follow:

- *Basic education level*: proportion of the population aged 6 years and older who attended or currently attending basic school (from elementary to junior high school).
- *Illiteracy*: proportion of the population aged 15 years and older who cannot read and write any one of the three languages; one Ghanaian language, English and French.
- *Religion*: Proportion of the population identified as Christian, Islam, Traditional and other religion or none religion.
- *Urbanisation by population size*: In Ghana a locality within a district with a population of ≥ 5,000 people was classified as urban, and less than 5,000 as rural.
- *Population density:* Population per square kilometre within the district.
- *Inter or intra-migration*: Information on place of birth and the non-Ghanaian population were used to identify intra-migration of the population within Ghana, and inter-migration across national boundaries.
- *Traditional (unimproved) housing* units: Proportion of households living in houses with the outer walls/roofing/floor materials made of traditional materials such as mud brick/earth, wood, bamboo, thatch/palm leaf, sandcrete/landcrete and stone.
- *Household Overcrowding index*: Computed from the sum of the five indicators consisting of population per dwelling, single room occupancy and sleeping room, average household size and households per dwelling.
- *Dependency ratio*: Number of dependents (child and old age) per 100 people undertaking paid employment.
- *Employment-to-population ratio* (EPR): Age-specific proportions of the population aged 15 years and over who undertook paid employment.
- *Household in Agriculture*: Proportion of households for which at least, one person in the household is engaged in any type of farming activity; crop farming, tree growing, livestock rearing and fish farming.
- *Household Insanitation Index*: The indicators included were the main source of drinking water, toilet and bathing facilities, and solid and liquid wastes disposal. The WHO/UNICEF Joint Monitoring Programme for Water Supply and Sanitation [18] standard method of classifying sanitation facilities and drinking-water sources as ‘improved (safe)” and “unimproved (unsafe) was used in this study. The indicators identified as unimproved (unsafe) sanitary conditions were combined as insanitation index to reflect relative degree in a district.

### Spatial statistical analyses

Malaria incidence rates were estimated followed by assessment of the spatial dependency within and between the health outcome (malaria) and risk factors (sociodemographic risk factors). Statistically significant risk factors were selected for the excess risk and conditioned choropleth maps.

### Incidence estimation and spatial weights

The crude district-level annual malaria incidence rates, *R*_*Mal*_ for the *i*-th district in the year *t* was estimated as

1 $$ {R}_{Mal_{it}}=\frac{X_{Mal_{it}}}{P_{it}}\times 10,000 $$

where${X}_{Mal_{it}}$ denotes the reported malaria case counts at the district *i (i= 1, 2, … ,* n =170*)* for the year *t (t = 2010, 2011, … , 2014)*, and*P*_*it*_ denotes the population in district *i* for the year *t.* The cumulative and five-year average incidence rates were also calculated for each district.

A spatial weights matrix was created based on first-order queen polygon contiguity. The effects of first-order queen polygon contiguity, merging both rook and bishop contiguities, are sufficient to capture spatial autocorrelation given the size and shape of the districts in Ghana. The irregularity of the shapes of the districts, hence the adoption of this contiguity approach in past studies to avoid neighbourless districts [19–21] and it is suitable to represent malaria transmission. Rook or bishop contiguity can leave gaps, which would not represent malaria transmission very well. Hence districts that shared common edges and/or common corners were considered neighbours and weights were assigned to these identified neighbours. The spatial weights were row-standardized such that for each row Σw_ij_ = 1 if districts *i* and *j* shared a common boundary; otherwise Σwij = 0, for non-neighbouring districts. Following standard convention, we excluded “self influence” by assuming that w_ii_ = w_jj_ = 0 so that *W* has zero diagonals.

### Empirical Bayes Smoothing of incidence rates

We used Empirical Bayes Smoothing using the principle of shrinkage [1, 22, 23] to stabilise incidence rates for areas with small populations or disease counts. We assumed that the relative risks of people residing in district *i*

(*δ*_*i*_) were independently and identically distributed according to a Poisson distribution:

2 $$ {x}_i/{\delta}_i\sim Poisson\left({N}_i{\delta}_i\right) $$

where *x*_*i*_ is the random variable representing disease count in district *i* while *N*_i_ is expected count for the same district. The Empirical Bayes Smoothed (EBS) relative risk of malaria, ${\widehat{R}}_{Mal_{it}}$ *borrows* the neighbouring district rates to adjust the uncertain rates as per the expression:

3 $$ {\widehat{R}}_{Mal_{it}}={\phi}_i{R}_{Mal_{it}}+\left(1-{\phi}_i\right){m}_{\delta_i} $$

where *ϕ*_*i*_ is the ratio of prior variance to the data variance, and ${m}_{\delta_i}$is the prior mean (weighted sample mean). The final EBS rate remains practically unchanged for districts with relatively large population or cases [23].

### Measuring spatiotemporal patterns and disease-risk factor associations

We checked and established spatiotemporal patterns of rates from 2010 to 2014 using global and local Moran’s indices. Global Moran’s I was used to determine whether or not identifiable spatial patterns exist over space and time [4, 5, 22] and Anselin local Moran’s I_i_ (the most widely used Local Indicator of Spatial Association, LISA), to identified specific districts and locations exhibiting spatial autocorrelation with their neighbouring districts as clusters or outliers [23–25]. The statistical inference was based on Monte Carlo randomisation test at 999 permutations with significance pseudo *p*-value<0.05 [4, 5, 19]. Non-spatial correlation was evaluated with Pearson correlation while global bivariate Moran's I was estimated to examine the spatial correlation between the five-year average incidence of malaria and the sociodemographic covariates. The statistically significant sociodemographic determinants were selected for excess risk and conditioned choropleth maps. Due to the ERM and CCM computational functionality in GeoDa, all spatial statistical maps were generated using GeoDa software version 1.12 even-though this package has lower cartographic quality as compared to other spatial packages especially ArcGIS.

### Mapping excess risk ratio as influenced by risk factors

The excess or relative risk is a form of standard morbidity or mortality rate (SMR) often used in public health which is estimated as the ratio of observed rate to the expected rate. The expected rate is the average rate for all the population at risk in each location which is computed as the ratio of the sum of all events in all locations to the sum of all the populations at risk [4, 5]. Implemented with excess risk map functionality in GeoDa, we calculated excess risk maps (ERMs) of malaria incidence (*event variable*) for each statistically significant socio-demographic covariate (*base variable*) [4, 5, 26].

### Exploring Malaria incidence with Conditioned Choropleth Maps

Both non-spatial Pearson correlation and spatial bivariate Moran's I analyses were performed between every pair of statistically significant risk factors to determine how they might act together or in sequence to influence malaria transmission. These analyses informed selection of the pairs of risk factors for the conditional choropleth mapping**.** We adopted conditioned choropleth mapping using the five-year average incidence rates of malaria as dependent variable (*theme variable*) and two strongly correlated significant sociodemographic factors (*covariates*) to visualise the three variables simultaneously. This resulted in a 3 x 3 panel of nine micromaps for which panel columns corresponded to the three categories of one covariate and the rows correspond to the three categories of the other covariate.

## Results

### Descriptive analysis and rates mapping

The minimum five-year average of 157 per 10,000 populations was observed for the capital city (Accra metropolis) of Ghana (Table 1). Sekyere East district recorded the greatest average incidence of malaria over the five-year period. The annual mean incidence of malaria in Ghana almost doubled during the study period from 996 per 10,000 in 2010 to 1,843 per 10,000 in 2014.

**Table 1:** Malaria incidence per 10,000 at-risk in Ghana for the period 2010 – 2014

Statistics	2010	2011	2012	2013	2014	Average rate
Min. (location)	4 (Sekyere central)	4 (Fanteakwa)	22 (Chereponi)	190 (Accra metro)	9 (Bosome Freho)	157 (Accra metro.)
Max. (location)	4396 (Bawku West)	4665 (Sunyani municipal)	12110 (Ahanta West)	6569 (Bawku West)	20120 (Sekyere East)	6473 (Sekyere East)
**Mean**	**996**	**1064**	**1300**	**1682**	**1843**	**1377**

**Fig. 2:**
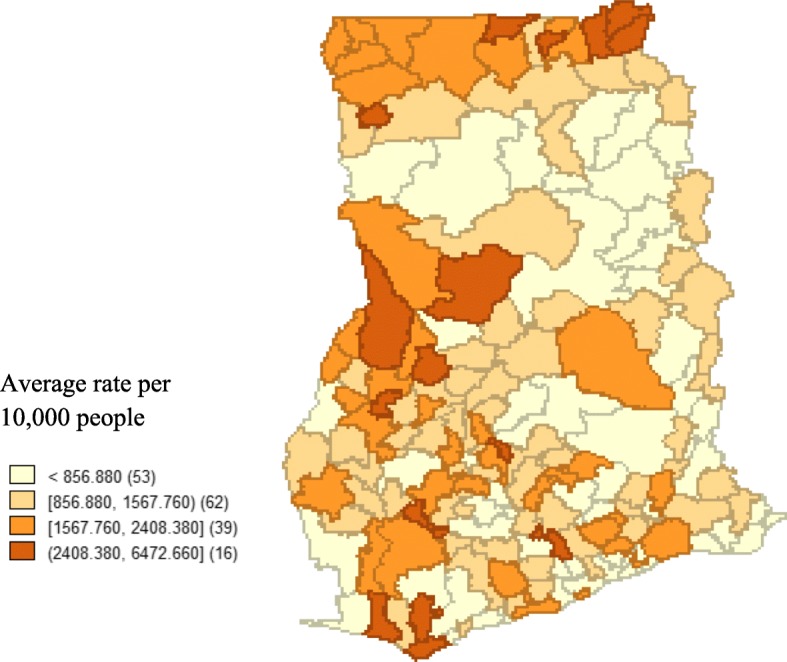
A 2010 – 2014 average incidence of malaria for the 170 districts in Ghana, grouped by five Jenks natural breaks. Numbers in the brackets indicate the number of districts for the rate ranges. The map was generated using GeoDa statistical software version 1.12

Natural breaks (Jenk’s) classification technique was used to identify categories of malaria incidence during the study period (Figure 2). Incidence of malaria varied geographically across the country with the greatest endemic districts located in the uppermost regions of the country and spread sparsely over the middle and southern belts.

### Spatial autocorrelation and cluster-outlier detection

Malaria incidence by district and year positively correlated with incidence in the neighbouring districts and the incidnece of the immediate previous year (Figure 5 in Appendix). This space-time association was strongest from 2012 to 2013 which exhibited the greatest Moran’s I and lowest pseudo *p*-value (<0.001). The local spatial maps indicated specific districts experiencing the high rates as compared to their neighbouring districts and further classify areas as clusters of high-high (called hotspot) and low-low (called coldspot) or outliers of low-high and high-low rates (Figure 6 in Appendix). On avearge, 143 out of the 170 districts had non-significant spatial clusterings while 7 and 14 locations were respectively identified as hotspot and coldspot clusters with the remaining 6 areas as outliers. The few districts with significant hotspots were located in the uppermost eastern part of the country. The results indicated that majority of the districts had malaria incidence rates not dissimilar from their neighbouring districts.

### Sociodemographic determinants and association with malaria incidence

Among the 13 risk factors analysed, 7 of them had significant spatial autocorrelation (Table 2 in Appendix). The risk factor most spatially autocorrelated was the proportion of people in traditional African religious practices (Moran's I = 0.0657, z = 10.855). Seven socio-demographic determinants correlated significantly with the incidence of malaria either spatially and/or non-spatially. Urbanisation exhibited only non-spatial correlation and intramigration exhibited only spatial correlation, while basic education, none/other religion, intermigration, employment-to-population and proportion of household into farming correlated with malaria incidence both spatially and non-spatially. With the exception of intermigration and proportion of household in agriculture, all the significant determinants correlated negatively with the incidence rate of Malaria infection. Specifically, increases in the proportion of the population attaining basic education associated with decreases in malaria incidence. Districts that were more urbanised had lower incidence of malaria. Districts with greater proportions of people aged 15 years and over who were employed had lower incidence of malaria. Compared to no religious affiliation, christian, traditional and islamic religions had lower malaria incidence. The strength of the correlations for all covariates were weak with spatial correlations weaker than the non-spatial correlations.

### Excess risks of malaria

**Fig. 3:**
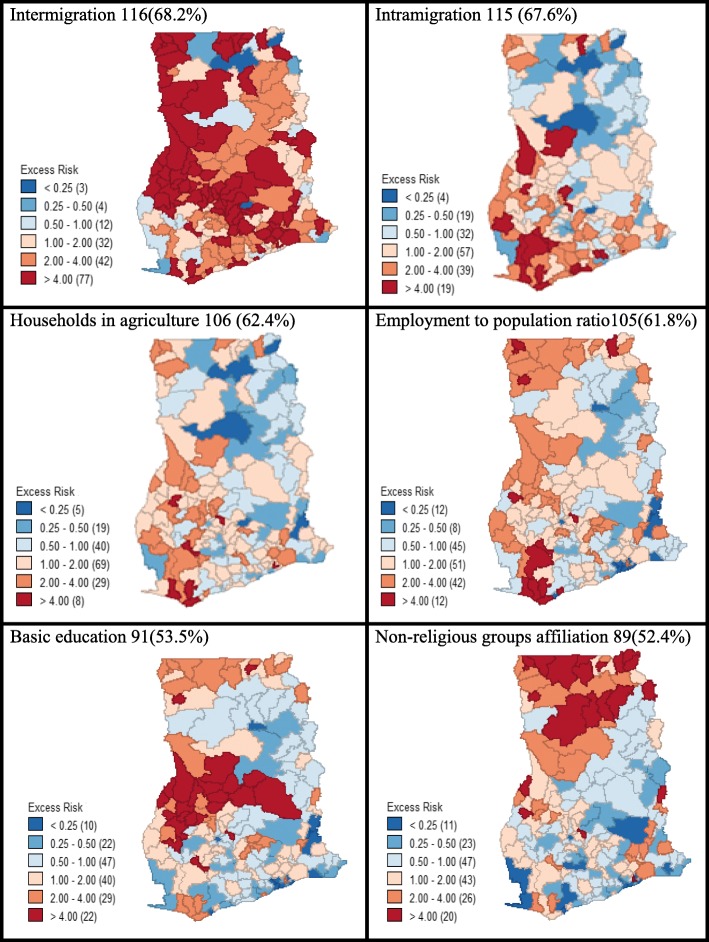
Distribution of excess malaria incidence in Ghana with respect to the statistically significant sociodemographic factors as base covariate, indicating the number and percentage of districts having malaria incidence greater than expected incidence. The colour codes indicate the relative or excess risk of each predictor variable and the numbers in the brackets represent the number of district. The maps were generated using GeoDa statistical software version 1.12

Excess malaria morbidity maps were created using the statistically significant sociodemographic factors as the base covariate and the five-year average incidence of malaria as event variable. We did not derive an excess risk map for malaria with respect to urbanisation because some districts (*N*=7) were completely rural (urban population <5,000). Consequently, all the six risk factors used for the malaria excess risk analysis (Figure 3) had spatial correlation with the incidence of malaria. A greater proportion (116; 68.2%) of locations had more than expected malaria incidence using intermigration as the base covariate and where as high as 77(45.3%) of the districts had elevated rates that were more than four times as the expected rate. The majority of the districts (115; 67.6%), mostly at the south-western parts of the country, experienced more than expected incidence of malaria as compared to the average rate across the entire study area when intramigration was imposed as the base covariate. The distribution of excess malaria incidence for agriculture located 106 (62.4%) districts with incidence greater than expected. For influence of the employment to population ratio, most of the locations (61.8%) experienced more than expected incidence of malaria as compared to the average rate in the whole study area. For basic education, a little above half, 91(53.5%) of the districts had greater than expected malaria incidence, almost all of them clustered in the middle part of the country. Upon considering non-affiliation to religious groups as determining factor of malaria morbidity across the study, slightly above half of the districts (89; 52.4%) had malaria incidence greater than expected, and clustering was pronounced at the northern belt.

### Correlation between risk factors and conditioned choropleth maps

The strongest positive non-spatial correlation (*p*< 0.01, *r* = 0.969) was observed between Urbanisation and Basic education attainment (Table 3 in Appendix). The strength of spatial correlations (bivariate Moran’s I) were generally lower than the non-spatial Pearson correlations.

**Fig. 4:**
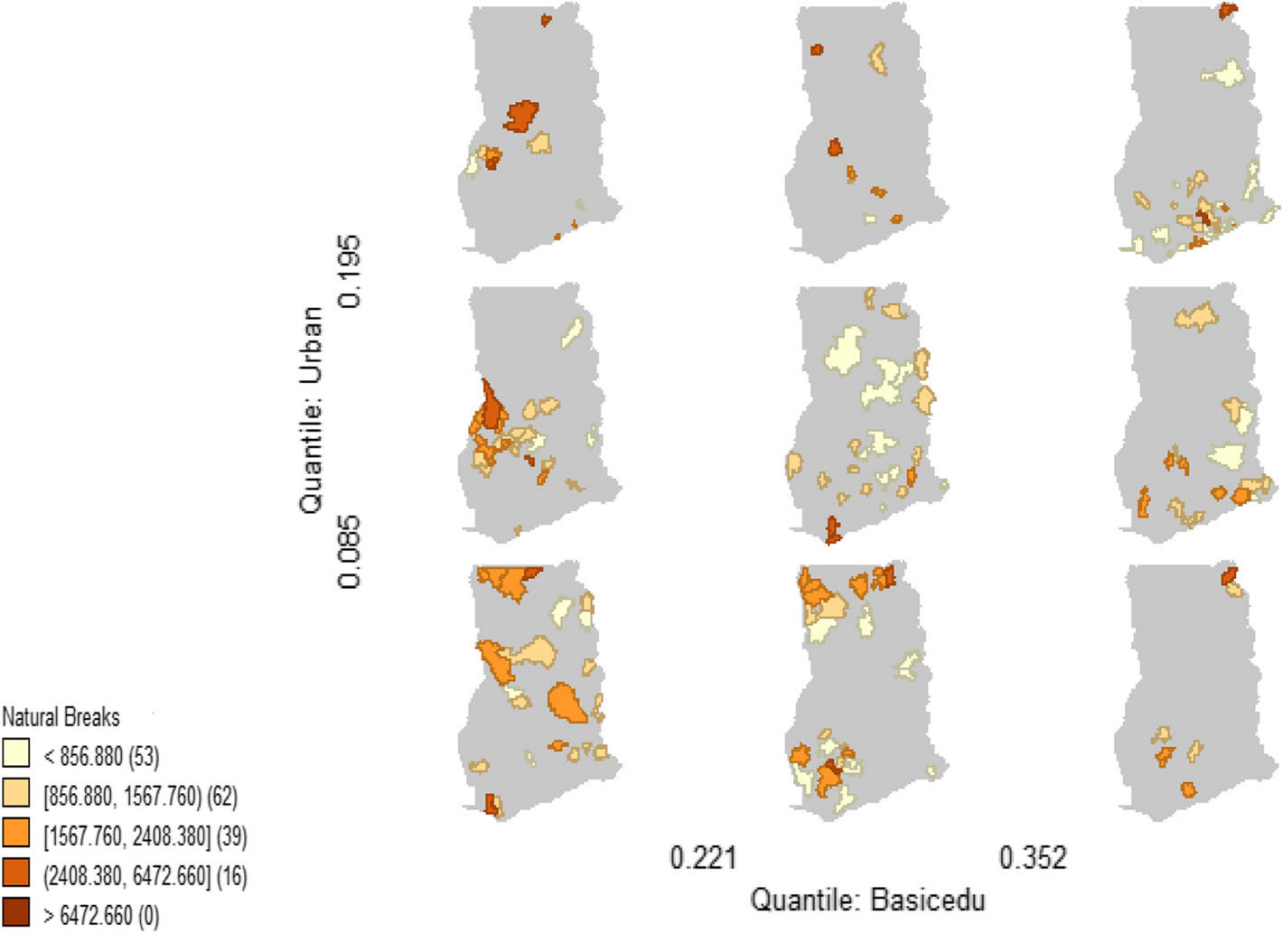
Malaria incidence conditioned on basic education-urban population where districts with relatively high rates due to co-location of the two risk factors were indicated by the different intensities of the brown colour. Numbers in the brackets indicate the number of districts for the rate as influenced by the two predictor variables. The maps were generated using GeoDa statistical software version 1.12

**Fig. 5:**
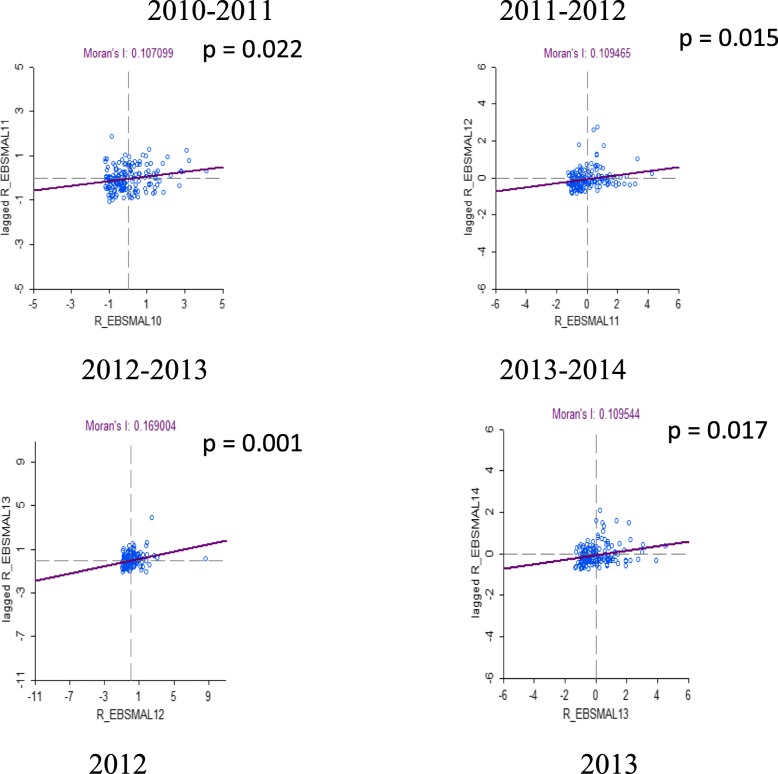
Bivariate scatter plot of the space-time correlation of malaria rates in the neighbouring districts and the incidnece of the immediate previous year. R-EBSMAL: Empirical Bayes’ Smoothed incidence rate of malaria. The maps were generated using GeoDa statistical software version 1.12

**Fig. 6:**
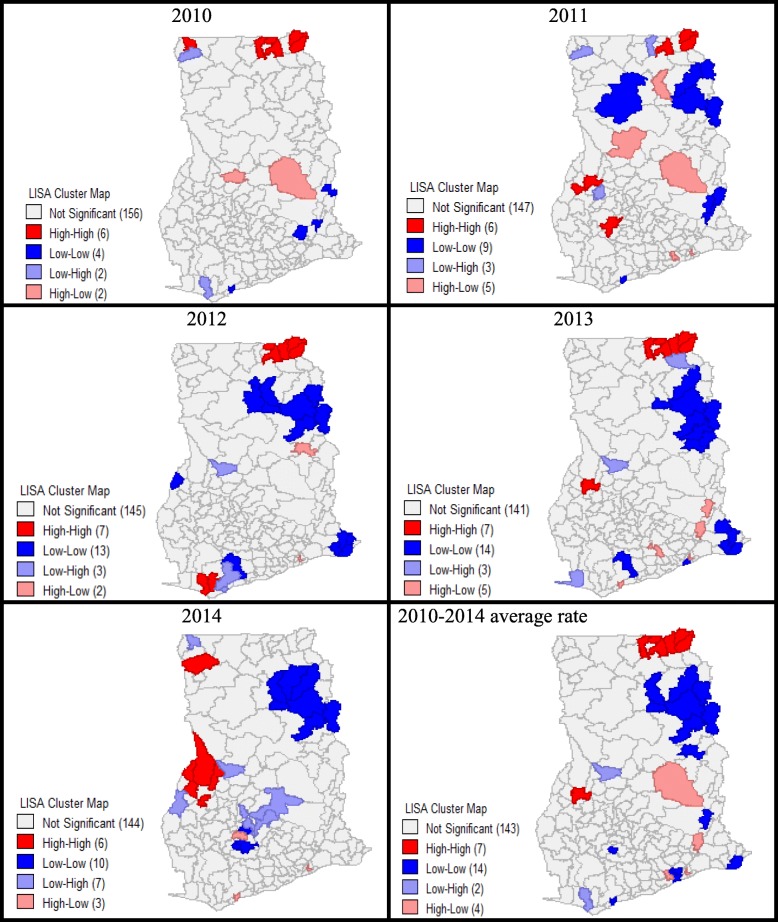
Local Moran’s I cluster-outlier maps of malaria incidence for the years 2010 to 2014 and the average incidence with statistical inference based on Monte Carlo randomisation test at 999 permutations, showing significant pseudo *p* < 0.05 clusters of High-High (hotspot) and Low-Low (colspot); and outliers High-Low and Low-High. LISA: Local Indicator of Spatial Association. The maps were generated using GeoDa statistical software version 1.12

**Fig. 7:**
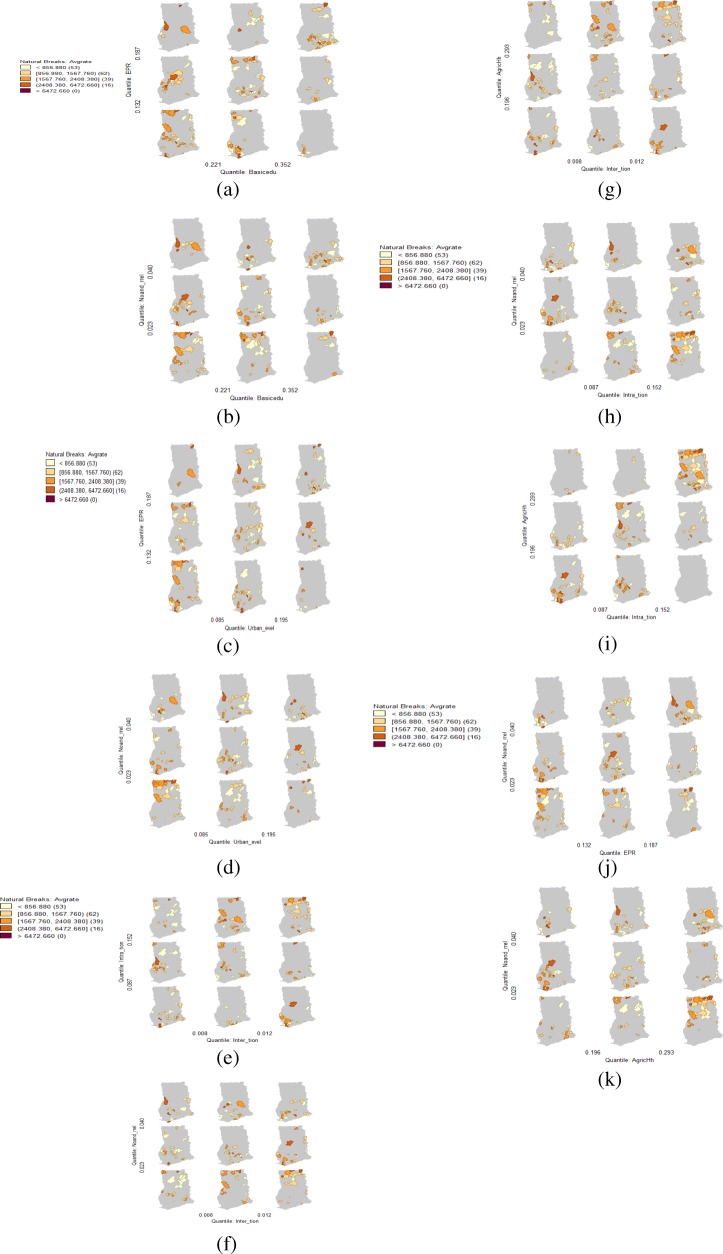
**a**, **b** Malaria incidence conditional on basic education and (**a**) employment-to-population ratio (**b**) no/other religion where districts with relatively high rates due to co-location of the two risk factors were indicated in the different intensities of the brown colour. Numbers in the brackets indicate the number of districts for the rate as influenced by the two predictor variables. The maps were generated using GeoDa statistical software version 1.12. **c**, **d** Malaria incidence conditioned on urban and (**c**) employment-to-population ratio, and (**d**) no/other religion where districts with relatively high rates due to co-location of the two risk factors were indicated by the different intensities of the brown colour. Numbers in the brackets indicate the number of districts for the rate as influenced by the two predictor variables. The maps were generated using GeoDa statistical software version 1.12. **e**, **f**, **g** Malaria rate conditional on intermigration and (**e**) intramigration, (**f**) no/other religion (**g**) agric households where districts with relatively high rates due to co-location of the two risk factors were indicated by the different intensities of the brown colour. Numbers in the brackets indicate the number of districts for the rate as influenced by the two predictor variables. The maps were generated using GeoDa statistical software version 1.12. **h**, **i** Malaria rate conditional on Intramigration and (**h**) No/other religion and (**i**) Agric households where districts with relatively high rates due to co-location of the two risk factors were indicated by the different intensities of the brown colour. Numbers in the brackets indicate the number of districts for the rate as influenced by the two predictor variables. The maps were generated using GeoDa statistical software version 1.12. **j**, **k** Malaria rate conditional on no/other religion and (**j**) Employment-to-population ratio (**k**) Agric household where districts with relatively high rates due to co-location of the two risk factors were indicated by the different intensities of the brown colour. Numbers in the brackets indicate the number of districts for the rate as influenced by the two predictor variables. The maps were generated using GeoDa statistical software version 1.12

The significantly correlated paired covariates were used as conditioning variables (horizontal and vertical variables) of the average malaria morbidity (response or theme variable) to construct the malaria conditional choropleth micromaps (CCMs). The districts with revealing multivariate spatial relationship were indicated in the brown colour code and the degree of malaria incidence was expressed by the intensity of the colour and categorized into five, indicating very low, low, moderate, high and very high rates. Considering urbanisation-basic education- malaria CCM (Figure 4), when both urbanisation and basic education attainment were high (top-right panel), the emerging districts with high rates of malaria were found for the southern districts but not as many as when urbanisation and basic education were low (bottom-left panel), which were detected at the northern districts. The lowest number of locations with emerging malaria incidence conditioned on urbanization and basic education were detected in the bottom right panel where basic education was high with low urbanization (more rural). The results of the nine micromaps for each multivariate relationship indicated geographical correlations and co-location among the three variables simultaneously where two conditioning risk factors were having co-occurrence effect on the incidence of malaria (Figure 7a-k in Appendix). Co-location of all of the covariates, even when both had low proportions (bottom-left micromap in the panels) or where both had high proportions (top-right micromap) filtered specific districts affected by overall high malaria incidence.

## Discussion

This was the first study to investigate the role of sociodemographic vulnerabilities and spatial variations of malaria incidence in Ghana using data with nationwide coverage of clinically confirmed cases from out-patient visits for all health facilities in the country. We applied excess risk and conditioned choropleth maps for visual display of geographic patterns in disease–risk factor interactions to simplify the understanding of spatial epidemiological data to health policy makers and public health practitioners.

### Malaria incidence and spatiotemporal trend

The incidence of malaria increased markedly between 2010 and 2014. Our findings show that malaria remained a prevalent public health threat in Ghana with increasing trend among the districts despite numerous interventions in the country as reported previously [21, 27]. The increasing trend in malaria incidence might also have been induced by increased access to health care, especially provision of more community health planning services and clinics and free treatment of malaria at health facilities through National Health Insurance Scheme (NHIS) which encouraged more people to visit health facilities for healthcare during the study period. Significantly clustering of rates was consistently high in the uppermost parts of the country but also shifted gradually through the middle to the southern belts. Thus as steps were implemented to control the risk at one location heterogeneously, risks moved to low endemic areas, possibly due to human and vector migrations. The implication of our findings is that short-term impacts on transmission intensity, following scaled up insecticide-treated net coverage will be more difficult to achieve in high transmission settings like Ghana compared to areas where the majority of the population have a lower intensity transmission profile [28]. It is well documented that malaria transmission intensity exhibits strong spatial heterogeneity even at a local level in highly endemic areas [12–14]. Despite few areas can be classified as relatively low risk areas (colspots), malaria incidence was generally high across the country with some districts experiencing relatively elevated burdens (hotspots). This finding is consistent with the climatic and environmental malaria risk factors analysis by Kumi-Boateng et al [29] and NMCP (28) in Ghana. Spatiotemporal clustering was relatively most observable in districts in the upper east region of the country.

### Association of malaria incidence with sociodemographic determinants

We found a substantial number of the sociodemographic risk factors having significant influence on the transmission dynamics of malaria in Ghana. The weak correlations of the sociodemographic risk factors with the incidence of malaria indicate that these factors might not be major predictors of the occurrence of malaria. The contribution of sociodemographics, such as the employment-to-population ratio, agricultural activities, might be explained by several inter-connected factors. The poor would generally find it very difficult to afford the necessary prevention and control interventions such as anti-malarial chemotherapy, mosquito coil and repellents, access to clean and mosquito-free breeding environments and good housing units. Additionally, populations with low socioeconomic status reside in rural areas and often engaged in farming activities which increase exposure to the vector, have relatively poorer education and knowledge about malaria prevention, are more marginalised, and have less access to quality healthcare for prompt diagnosis and treatment. This also affects the health seeking and treatment behaviour in poorer rural areas [30] that can serve as reservoirs of the *Plasmodium* sp. parasite for the vector transmission. Housing the undiagnosed and untreated malaria parasites could facilitate the transmission through intra and inter-migrations and high mobility of the mosquito vector into low endemic areas or areas with lower levels of immunity [13]. Hence low socioeconomic status, limited education, low levels of urbanization, high migration and agricultural levels associated with greater levels of malaria morbidity across the local districts. Thus successes for malaria control also depend significantly upon knowledge, sociodemographic and socioeconomic status of the affected populations in endemic countries [15, 28] in addition to the biologic, climatic and environmental factors. In hyperendemic settings of malaria, the disease tends to spatially cluster based on different levels of environmental and climatic conditions and may also cluster with the sociodemographic factors [26]. One unexpected finding which is not well documented in literature is the influence of non-affiliation with religious groupings. Although the three main religious stratifications in Ghana did not correlate significantly with the incidence of malaria, non-religious affiliations significantly correlated inversely. The religious affiliations where people often come together and even stay overnight for prayers and other religious activities and at times in open spaces and some surrounded by bushes and in forest will expose people to the vector and those who might be having asymptomatic/ undiagnosed and untreated malaria. This means that targeted risk management strategies are needed for religious (Christianity, African traditional and Islamic) gatherings.

### Visual display of geographic patterns in malaria incidence–risk factor interactions

The ERMs and CCMs identified specific locations of high incidence that could be targeted [31] where malaria and specific sociodemographic factors were spatially interacting significantly. It is plausible that some of these sociodemographic factors could inform the management of the incidence of malaria in the identified districts. For instance, spatial interaction of malaria-basic education specifically highlighted districts in the middle and northern parts where increasing basic education and/or intensifying education campaign could contribute to reducing the expected incidence of malaria. Improving economic conditions in those districts (which will impact migration) could improve health seeking behaviour of the indigenes to help lower malaria incidence risk [13] in those districts. The ERMs revealed specific districts requiring extra attention that could be targeted for modifying a particular sociodemographic covariate in an attempt to lower the incidence of malaria, mostly at the northern and south-western parts. The conditioned micromaps of malaria located areas that are most/least affected of malaria with low and/or high accumulation of two risk factors. Our findings imply that although multiple factors are associated with malaria morbidity, none can be isolated as a sole target for malaria control. However, the ERMs and the CCMs highlighted specific locations that can be targeted for particular or pair-wise combinations of sociodemographic factors to address the spatial heterogeneous transmission of malaria. Due to the complex interactions observed in the risk factors and disease-risk factor spatial interactions, targeting each or pair could lead to extended or multiple control effects on malaria transmission. Specifically, improving employment-to-population ratio in rural areas will increase education attainment for improve knowledge and healthy lifestyle, increase the ability to afford good healthcare and good housing facilities, reduce rural-urban migration which in turn reduces urban slums with its associated health implications. These chain effects will significantly impact the successes for malaria control, especially in endemic areas.

## Limitations

The study used aggregated data collected by health professionals on patients at health facilities.

Individual-level information was not available and hence the result should be interpreted cautiously to avoid ecological fallacy. This study comprehensively assessed all cases of malaria registered at health facilities, but we cannot rule out under ascertainment of malaria due to variable levels of access to care. This study did not include some known risk factors of malaria (biologic, environmental and climatic factors) which might have major impact on transmission of malaria and we did not have information on district-level information on the coverage and usage of the intervention programmes.

## Conclusion

The findings of this study indicated that Ghana still remains hyperendemic region of malaria, generally substantial rates in all districts with spatiotemporal dynamics, indicating influence of malaria incidences from close-connected districts and previous years. This study reaffirms the need to improve access to healthcare and malaria intervention programmes across the country, while providing more weight to certain districts. We found and identified areas where each and combinations of sociodemographic risk factors resulted in different geographic clusters of malaria incidence. The significant sociodemographic risk factors to be considered or/and improved in the development and implementation of malaria control programmes are increasing at least basic education, reducing accumulation of people at a place (urbanization) through provision of socio-economic opportunities (e.g; increase employment-to-population ratio) in rural areas to reduce intra and inter-migration, educating rural folks in the agriculture, and grouping of people for religious practices. With respect to the volume of information appearing in a single geo-visual display, the risk factor-linked-rate maps provide more opportunities for detailed and efficient epidemiological assessments than separate rate or risk thematic maps. The ERMs and CCMs made it possible to filter geographical areas heavily affected with the disease with accumulation of specific risk factor(s). We found that applications of ERMs and CCMs visually displayed and identified specific areas with emerging disease–risk factor interactions for informed, improved or prioritized malaria interventions. Spatial regressions such as spatial lag and spatial error models are best for establishing significant spatial associations but cannot visually display and specifically pinpoint where these associations are significant. Hence in addition to sophisticated spatial regression models, the easily interpretable ERMs and CCMs should be used to identify the locations of the significant disease-risk factors associations, simplifying complex spatial epidemiological information to health policy makers and public health practitioners who might not be experts in spatial statistics. Moreover, due to the ease implementation with open access GeoDa spatial software, ERMs and CCMs could become part of the routine reporting and monitoring approach to facilitate health disparities assessment and future health outcome predictions for improved health intervention programmes.

## Appendix

**Table 2:** Global spatial autocorrelation of risk factors and their correlation with malaria incidence

Risk factors	Global spatial autocorrelation			^#^Pearson's correlation with outcome rate		Bivariate spatial correlation with outcome rate		
	Univariate Moran's I	Pseudo *p*-value	z-value	Pearson's r	*p*-value	Bivariate Moran's I	Pseudo *p*-value	z-value
Basic education	0.138***	0.019	3.193	-0.226*	0.003	-0.062***	0.042	-1.493
Illiteracy	0.013	0.056	1.650	-0.103	0.180	-0.0004	0.465	-0.069
Religion								
Christian	-0.009	0.275	-0.072	-0.057	0.457	-0.019	0.329	-0.491
Muslim	0.277***	0.002	5.186	-0.137	0.075	-0.039	0.140	-0.971
Traditional	0.657***	0.001	10.855	0.014	0.856	0.019	0.324	0.414
None/Other	0.176***	0.010	3.671	-0.194*	0.011	-0.064***	0.046	-1.505
Urban lev.	0.117***	0.022	2.860	-0.174*	0.023	-0.053	0.070	-1.287
Insanitary lev.	0.018	0.080	0.881	-0.002	0.983	0.008	0.374	0.226
Intermigration	-0.001	0.125	0.828	0.184*	0.016	0.089***	0.038	1.969
Intramigration	0.102***	0.034	2.100	0.144	0.060	0.111***	0.020	2.402
Traditional housing unit	0.030	0.129	0.849	-0.122	0.112	-0.021	0.314	-0.523
Household overcrowding index	0.076***	0.031	2.112	-0.148	0.054	-0.034	0.190	-0.836
Pop. density	0.018	0.080	0.881	-0.002	0.984	0.008	0.374	0.227
Dependency ratio	0.550***	0.001	9.051	-0.020	0.795	0.070	0.050	1.627
Employment to-population ratio	0.185***	0.012	4.777	-0.189*	0.014	-0.061***	0.042	-1.503
Agric household	0.122***	0.021	2.8055	0.178*	0.020	0.115***	0.015	2.536

* significance level at *p*< 0.05 (2-tailed); ** significance level at *p*< 0.01 (2-tailed)

*******spatial significance level at pseudo *p* < 0.05 for conditional 999 permutations.

# Spatial correlation is most appropriate but the spatial correlation was much smaller than the non-spatial correlation and the departure from independence is consistent but weak. The assumption of spatial independence may have affected the non-spatial correlation result.

**Table 3:** Non-spatial correlation (Pearson) and spatial correlation (Bivariate Moran’s I) between pairs of statistically significant covariates for districts in Ghana

Pairwise risk factors	^#^Non-spatial Pearson's correlation		Spatial Bivariate correlation	
	r	*p*-value	Moran's I	*p*-value
Basic edu-Intermigration	0.001	0.992	0.032	0.064
Basic edu-Intramigration	-0.039	0.610	-0.003	0.373
Basic edu-Urban	0.969**	0.000	0.113*	0.023
Basic edu-Emp.-to-pop. ratio	0.925**	0.000	0.145*	0.018
Basic edu-No/other religion	0.861**	0.000	0.094*	0.025
Basic edu-Agric household	-0.014	0.854	0.013	0.198
Intermigration-Urban	-0.028	0.719	-0.008	0.404
Intermigration- Intramigration	0.612**	0.000	0.028	0.062
Intermigration-Emp.-to-pop. ratio	-0.011	0.888	-0.0005	0.240
Intermigration-No/other religion	-0.046	0.552	-0.046*	0.043
Intermigration-Agric household	0.867**	0.000	0.030*	0.047
Intramigration-Urban	-0.052	0.497	-0.0209	0.288
Intramigration-Emp.-to-pop. ratio	-0.040	0.607	-0.015	0.418
Intramigration-No/other religion	-0.084	0.275	-0.069*	0.007
Intramigration-Agric household	0.915**	0.000	0.115*	0.025
Urban-Emp.-to-pop. ratio	0.941**	0.000	0.145*	0.019
Urban-No/other religion	0.860**	0.000	0.098*	0.026
Urban-Agric household	-0.050	0.521	-0.034	0.059
Emp.-to-pop. ratio- No/other rel.	0.835**	0.000	0.104*	0.022
Emp.-to-pop.ratio-Agric household	-0.030	0.693	-0.015	0.388
No/other rel.-Agric household	-0.075	0.331	-0.128*	0.001

** Non-spatial correlation is significant at *p*< 0.01 (2-tailed)

*****spatial correlation is significant at pseudo *p* < 0.05 for conditional 999 permutations

# Spatial correlation is most appropriate but the spatial correlation was much smaller than the non-spatial correlation and the departure from independence is consistent but weak. The assumption of spatial independence may have affected the non-spatial correlation result

## Abbreviations

ERM: Excess Risk Map
CCM: Conditional/Conditioned Choropleth Map
EBS: Empirical Bayesian smoothed
GHS: Ghana Health Service
GSS: Ghana Statistical Service
ITN: Insecticide-treated Nets
KNUST: Kwame Nkrumah University of Science and Technology
NMCP: National Malaria Control Programme
PHC: Population and Housing Census
SMR: Standard Morbidity/Mortality Rate
WHO: World Health Organisation
